# Bidirectional Mendelian randomization analysis reveals no causal association between Helicobacter pylori infection and osteoporosis risk

**DOI:** 10.1097/MD.0000000000045185

**Published:** 2025-10-31

**Authors:** ZhiXiang Chen, Xiao Yang, YaoWu Chen, LuYao Wang, MengLi Ji, MaoWen Wang, JianMin Fan, Wen Zhang

**Affiliations:** a First Affiliated Hospital of Hunan University of Traditional Chinese Medicine, Changsha City, Hunan Province, China; b Hunan University of Traditional Chinese Medicine, Changsha City, Hunan Province, China.

**Keywords:** Bidirectional Mendelian randomization analysis, causal association, *Helicobacter pylori* infection, osteoporosis

## Abstract

Despite the widespread controversy surrounding the existence of a causal link between contracting *Helicobacter pylori (H pylori*) and the risk of osteoporosis (OP), this study aimed to ascertain this link using bidirectional two-sample Mendelian randomization (MR) analysis powered by genome-wide association data. Various analysis methods were used, including the inverse variance-weighted (IVW) method, weighted model, simple model, MR-Egger regression, and the weighted median method. The MR-Egger regression intercept term, MR-PRESSO, Cochran *Q* test, and leave-one-out cross-validation technique were used for sensitivity analysis. The IVW method of two-sample MR showed no causal relationship with anti-*H pylori* IgG seropositivity and OP (OR: 1.03; 95% CI: 0.90–1.18; *P* = .69), and the level of *H pylori* vacuolating cytotoxin A (VacA) antibody (OR: 1.00; 95% CI: 1.00–1.00; *P* = .28) or *H pylori* cytotoxin-associated gene A protein (CagA) antibody level (OR: 0.95; 95% CI: 0.86–1.05; *P* = .31). Using MR, a bidirectional analysis revealed no causal association between *H pylori* infection and OP. This finding was consistent across the 4 complementary analytical methods, and the sensitivity analyses identified no violations of the underlying MR assumptions. Our results suggest that there is no evidence of a causal relationship between *H pylori* infection and OP.

## 1. Introduction

Osteoporosis (OP) is a systemic metabolic bone disorder characterized by declining bone mass, weakened bone tissue microstructure, increased bone fragility, and an increased risk of fractures.^[1]^ These osteoporotic fractures are the most severe complications of the disease, significantly increasing the risk of disability and even death upon occurrence, thus seriously endangering patients’ lives, health, and overall well-being and imposing a heavy burden on society and families. As the global population ages, OP has become a growing threat to the health of middle-aged and older individuals. By 2010, approximately 0.158 billion individuals were afflicted with OP globally, and this figure is expected to exceed 0.3 billion as of 2040.^[2]^ In the U.S., the prevalence of OP increased among adults aged ≥ 50 years, transitioning from 9.4% during 2007 to 2008 period to reaching 12.6% by the end of the 2017 to 2018 timeframe.^[3]^ In China, the medical costs for treating major osteoporotic fractures (wrists, vertebrae, and hips) are expected to surpass 130 billion yuan by 2035, and 160 billion yuan by 2050. Given its profound impact and escalating prevalence, the early diagnosis and prevention of OP are imperative.

*Helicobacter pylori (H pylori*), an acid-tolerant gram-negative bacterium, is widely recognized as the dominant species that commonly colonizes the stomach lining and is one of the most critical microorganisms in the human stomach. Currently, over half of humanity is infected with this bacterium,^[4]^ indicating that it is widespread worldwide. *H pylori* cytotoxin-associated gene A protein (CagA) and vacuolating cytotoxin A (VacA) are the primary virulence factors of Hp, which is one of the major pathogenic factors of gastritis, peptic ulcers, and cancer. Additionally, Hp infection may cause autoimmune, cerebrovascular, and cardiovascular diseases. These findings indicate that *H pylori* infections may have widespread and profound adverse effects on human health.

However, the association between *H pylori* infection and OP remains unclear. While early research hinted at a significant correlation, suggesting that *H pylori*-induced gastrointestinal inflammation and its associated products (such as ILs and TNF-α) might influence bone turnover and cause OP,^[5]^ other studies have proposed the opposite view, suggesting that there is no causal connection between *H pylori* infection and OP. This perspective highlights the complexity and limitations of these studies, mainly because most of the relevant studies were observational, with multiple potential factors that could interfere with the results. Inflammatory diseases such as chronic gastritis can confuse genetic associations by affecting *H pylori* infection and susceptibility to OP.

Mendelian randomization (MR) is an analytical technique based on the use of genetic variants (usually single nucleotide polymorphisms [SNPs]) as instrumental variables (IVs) to systematically and precisely analyze the causal relationships between exposure factors and diseases.^[6,7]^ Mendel independent allocation rule asserts that genetic variations are typically independent of each other and are not influenced by other factors or confounding variables. As a result, the findings of MR research are highly reliable.^[8]^ Our study conducted a meticulous two-sample bidirectional MR analysis to explore the possible causal connection between *H pylori* infection and OP, drawing upon public data from genome-wide association studies (GWAS).

## 2. Materials and methods

### 2.1. GWAS summary data

GWAS datasets for the anti-*H pylori* IgG, anti-*H pylori* VacA, anti-*H pylori* CagA and OP genes were obtained from an open-access GWAS database maintained by the Bristol University Integrative Epidemiology Unit (http://gwas.mrcieu.ac.uk/datasets/). The IgG dataset included 4683 samples and 7,247,045 SNPs. The VacA dataset comprised 1571 samples and 9,178,635 SNPs. The CagA dataset contained 985 samples and 9,165,056 SNPs. The OP dataset was obtained from the Finnish Alliance, and comprised 3203 cases, 209,575 controls, and 16,380,452 SNPs. All data samples originated from the European population, potentially mitigating population stratification bias to some extent. The details of the dataset are presented in Table 1.

**Table 1:** Detailed information on GWAS included in Mendelian randomizations.

Exposure/outcome	Population	Consortium	Population	URL
Anti-*H pylori* IgG levels	4683	EBI	European	https://opengwas.io/datasets/ieu-b-4905
*H pylori* VacA antibody levels	985	EBI	European	https://opengwas.io/datasets/ebi-a-GCST90006911
*H pylori* CagA antibody levels	1571	EBI	European	https://opengwas.io/datasets/ebi-a-GCST90006916
OP	3203	Finn	European	https://opengwas.io/datasets/finn-b-M13_OSTEOPOROSIS

EBI = European Bioinformatics Institute, GWAS = genome-wide association studies, *H pylori* = *Helicobacter pylori*, OP = osteoporosis.

### 2.2. MR design

**Figure 1.**
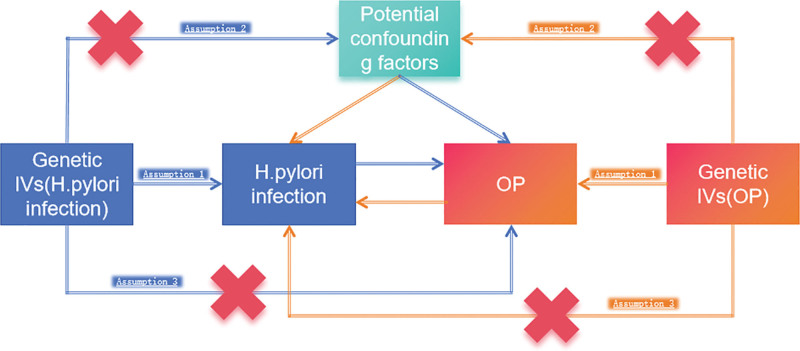
The 3 crucial hypotheses of the Mendelian randomization study are as follows: Assumption 1: genetic variants directly affect the risk factor; Assumption 2: the IVs should not be correlated with potential confounding factors; Assumption 3: the IVs should affect osteoporosis risk only through *H pylori* infection, not through other pathways. IVs = instrumental variable, *H pylori = Helicobacter pylori*, OP = osteoporosis.

The MR was used to assess the potential effect of *H pylori* infection on the risk of OP, and a reverse MR study was performed to investigate the potential effect of OP on the risk of *H pylori* infection. Utilizing a bidirectional two-sample MR model, illustrated in Figure 1, as its design framework, the researchers rigorously adhered to 3 basic principles of MR analysis:^[9]^ correlation hypothesis: there is a significant association between IVs and exposed factors; independence hypothesis: there are no confounding factors in the correlation between IVs and exposed outcomes, ensuring no related genetic polymorphisms; and exclusion hypothesis: IVs do not directly affect the results but can only be influenced by exposure factors.

### 2.3. Selection of the genetic instruments

To identify IVs, we systematically screened for SNPs that exhibited significant associations with exposure within the GWAS dataset using the following steps: First, we set a correlation limit of *P* < 5 × 10^−6^ to screen for SNPs that were genetically significantly linked to the exposed factors. Parameters *r*^2^ = 0.001 and kb = 10,000 were set to eliminate the cascading imbalance. We then extracted data on the exposed factors in the outcome and merged the exposure and outcome data while removing palindromic SNPs. Finally, to reduce the bias of low-strength IVs, the following formula was used to calculate the *F*-statistic for each SNP^[10]^


$$\begin{array}{l} F=\frac{N-2}{1-R^{2}} \end{array}$$

During the selection of the IVs, *F*-statistics served as a pivotal metric for assessing their strength. IVs with *F*-statistics < 10 were deemed weak and excluded, whereas those with *F*-statistics > 10 were retained as sufficiently strong for inclusion in the final MR analysis.^[11]^

### 2.4. Statistical analysis

We used the inverse variance-weighted (IVW) technique^[12]^ as our primary analytical approach, supplemented by an ensemble of additional techniques, such as MR-Egger regression, simple mode, weighted mode, and weighted median, to determine the potential bidirectional causal relationship between *H pylori* infection and OP. To further ensure the robustness of our data analysis, we used Cochran *Q* test to examine the heterogeneity of IVs. Heterogeneity was indicated by a *P*-value of < .05. To explore whether each SNP has horizontal pleiotropy, that is, whether SNPs may affect OP through pathways other than the direct pathway,^[13]^ the MR-Egger regression intercept test was employed, with a threshold of significance set at *P* < .05, to establish the presence of horizontal pleiotropy.^[14]^ Additionally, the presence of outliers was tested using the MR-PRESSO approach. Additionally, the leave-one-out method was used to screen for critical SNPs that significantly influenced the results. The statistical power of the experiments was calculated using the https://shiny.cnsgenomics.com/mRnd/ website.^[15]^ All statistical analyses were carried out using the TwoSampleMR 0.5.7 software package in RStudio (version 4.3.2; R Foundation for Statistical Computing, Vienna, Austria). The TwoSampleMR package is publicly available from the Comprehensive R Archive Network and was developed by the MRC Integrative Epidemiology Unit, University of Bristol, Bristol, United Kingdom.

### 2.5. Ethics statement

This study used existing secondary data from public databases for analysis. No further ethical clearance was mandatory because the data sources were publicly available and not collected for the first time.

## 3. Results

### 3.1. The outcome of H pylori infection and subtypes on OP

We selected 12, 15, and 15 SNPs from GWAS data for anti-*H pylori* IgG, *H pylori* VacA, and *H pylori* CagA antibodies. These SNPs were merged with the extracted OP data. After removing the palindromic SNPs, no outliers were detected in the MR-PRESSO test. The calculated *F*-statistic values of all the IVs exceeded 20, suggesting that there were no weak IVs. Finally, 11, 9, and 7 IVs were included in the MR analysis after meeting stringent selection criteria. Tables S1 to S3 (Supplemental Digital Content, https://links.lww.com/MD/Q338) list the IVs for *H pylori, H pylori* VacA, and *H pylori* CagA antibodies, respectively. Table 2 presents the results of the 5 statistical methods. The IVW technique indicated a lack of causal association between the anti-*H pylori* IgG (OR: 1.03; 95% CI: 0.90–1.18; *P* = .69), *H pylori* VacA antibodies (OR: 1.00; 95% CI: 1.00–1.00; *P* = .10), and *H pylori* CagA antibodies (OR: 0.95; 95% CI: 0.86–1.05; *P* = .31), and the risk of OP. All 4 supplementary methods reached the same conclusions. The Cochrane *Q* test did not reveal any heterogeneity, and the MR-Egger regression intercept analysis confirmed the absence of horizontal pleiotropy (Table S5, Supplemental Digital Content, https://links.lww.com/MD/Q338).

**Table 2:** Estimated bidirectional causal relationship between contracting *Helicobacter pylori* and osteoporosis through various MR techniques.

Exposure		*H pylori*	*H pylori* VacA	*H pylori* CagA	Osteoporosis
Outcome		Osteoporosis	Osteoporosis	Osteoporosis	*H pylori*
No. of SNPs		11	9	7	19
MR-Egger intercept *P*-value		.82	.82	.15	.13
*R*^2^ (%)		5	13	2245	468
*F*-statistics		224.95	207.57	225.92	234.55
MR-Egger	OR (95% CI)	1.07 (0.76,1.49)	1.00 (1.00,1.00)	1.19 (0.90,1.56)	1.20 (1.00,1.45)
	*P*-value	.72	.51	.27	.07
Weighted median	OR (95% CI)	1.07 (0.89,1.28)	1.00 (1.00,1.00)	1.01 (0.88,1.15)	1.06 (0.94,1.19)
	*P*-value	.49	.91	.92	.37
IVW	OR (95% CI)	1.03 (0.90,1.18)	1.00 (1.00,1.00)	0.95 (0.86,1.05)	1.05 (0.96,1.14)
	*P*-value	.69	.28	.31	.31
Simple mode	OR (95% CI)	1.07 (0.83,1.38)	1.00 (1.00,1.00)	1.01 (0.84,1.22)	1.02 (0.84,1.24)
	*P*-value	.61	.98	.89	.85
Weighted mode	OR (95% CI)	1.06 (0.82,1.37)	1.00 (1.00,1.00)	1.03 (0.87,1.21)	1.04 (0.88,1.23)
	*P*-value	.67	.99	.77	.68

CagA = cytotoxin-associated gene A protein, CI = confidence interval, *H pylori* = *Helicobacter pylori*, IVW = inverse variance-weighted, MR = Mendelian randomization, OP = osteoporosis, OR = odds ratio, SNPs = single nucleotide polymorphisms, VacA = vacuolar cytotoxin A.

### 3.2. The outcome of OP on H pylori infection

**Figure 2.**
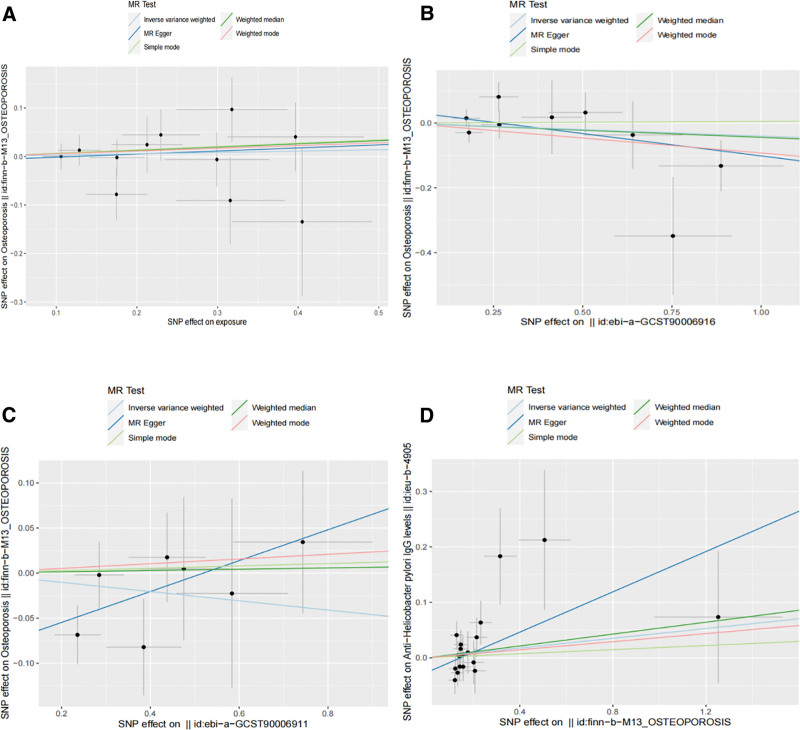
Scatter plot illustrating causal connections between *Helicobacter pylori* infection and osteoporosis using various MR techniques. (A) Causal estimates of *Helicobacter pylori H pylori* seroprevalence in OP patients. (B) Causal estimates of VacA expression in OP. (C) Causal estimates of CagA in the OP. (D) Causal estimates of OP in *Helicobacter pylori H pylori* infection. The inclination of each line represents the estimated causal effects determined using each method. OP = osteoporosis, VacA = vacuolating cytotoxin A.

We selected 22 significant SNPs from the OP GWAS data and merged them with anti-*H pylori* IgG dataset. After removing a single palindromic SNP, no outliers were identified during MR-PRESSO analysis. The calculated *F*-statistic values exceed 20, confirming the absence of weak IVs. Ultimately, 19 SNPs (Table S4, Supplemental Digital Content, https://links.lww.com/MD/Q338) were included in the MR analysis of the OP and anti-*H pylori* IgG. The conclusions reached by the 5 distinct statistical approaches were aligned, indicating no correlation between OP and the incidence of anti-*H pylori* IgG. The IVW results were (OR: 1.00; 95% CI: 1.00–1.00; *P* = .10), with no heterogeneity or horizontal pleiotropy (Table S5, Supplemental Digital Content, https://links.lww.com/MD/Q338). Figures S1 to S3 (Supplemental Digital Content, https://links.lww.com/MD/Q337) show leave-one-out plots, forest plots, and funnel plots of this study, respectively, to intuitively illustrate the effects of each SNP. Figure 2 shows scatter plots of the current study.

## 4. Discussion

This study explored the potential causal relationship between *H pylori* infection and OP using MR analysis of large-scale GWAS data. The results clearly show no causal relationship between *H pylori* infection and OP.

There is widespread controversy in the medical community regarding the potential association between *H pylori* infections and OP. Previous research has supported an association between contracting *H pylori* infection and OP. Pan et al^[16]^ conducted a retrospective analysis of health examination records from 867 Taiwanese participants, and the results revealed an independent and noteworthy association (OR: 1.09; 95% CI: 1.08–1.11, *P* < .001) between *H pylori* infection and bone mineral density among participants matched for age. In a more extensive cohort study involving 10,482 female researchers, Kim et al^[17]^ found that *H pylori* infection was associated with an elevated risk of OP. Similarly, a prospective cohort study by Luigi Gennari et al^[18]^ reported that contracting *H pylori* subtypes expressing the CagA protein is an essential factor that increases the risk of OP and fractures (OR, 1.31; 95% CI: 0.86–2.02, *P* = .211). A systematic review and meta-analysis of 21 studies by Wang et al^[19]^ further supported these findings, showing a significant link between *H pylori* infection and increased clinical risk of OP (OR, 1.39; 95% CI: 1.13–1.71; *P* = .021). Several explanations have been proposed to explain this association. First, contracting *H pylori* has the potential to induce a systemic inflammatory response, and the released cytokines such as IL-1, IL-6, and TNF-α may have an indirect effect on bone turnover. These inflammatory responses may lead to bone loss, decreased bone mineral density, and an increased fracture risk. Second, the decrease in vitamin B12 levels associated with *H pylori* infection may adversely affect bone remodeling. Furthermore, *H pylori* infection may cause gastric mucosal atrophy, which can affect calcium absorption.

However, other studies have provided evidence contradicting this view and supporting our findings. A study by Ye et al^[20]^ involved health examinations of 1388 individuals aged 50 years and above, and the findings indicated that *H pylori* infection does not act as an independent risk factor for the development of osteopenia or OP (OR = 1.31; 95% CI: 0.86–2.02; *P* = .211). Similarly, in their retrospective cross-sectional analysis, Huang et al^[21]^ found no correlation between *H pylori* serum positivity and total bone density, suggesting that *H pylori* infection may not be a crucial determinant of bone density loss (β = 0.006, 95% CI: −0.003 to 0.015; *P* = .177). Moreover, an additional cross-sectional investigation by Chinda et al^[22]^ involving 268 healthy men undergoing general physical examinations also found no association between contracting *H pylori* infection and reduced bone density.

Several factors contribute to the ongoing debate regarding the association between *H pylori* infection and OP. First, most of these studies were observational, deficient in blinding and randomness, and susceptible to unmeasured and unnoticed biases and confounding factors. Second, diagnostic methods for *H pylori* infection vary. The Urea Breath Test is the best noninvasive diagnostic method for *H pylori* infection, as recommended by the World Gastroenterology Organization Global Guidelines.^[23]^ However, in some studies, *H pylori* infection was diagnosed based on *H pylori* serology, which may not be sufficiently accurate. Third, the prevalence of *H pylori* infection varied geographically, which may have affected the results. In a meta-analysis, Hooi et al^[24]^ found that the infection rate of *H pylori* was highest in Africa (79.1 %), followed by Asia (54.7 %), Europe (47 %), and North America (37.1 %). An extra layer of research evidence comes from a meta-analysis revealing a considerable correlation between contracting *H pylori* infection and the risk of OP in 3 specific East Asian countries: China, Japan, and South Korea.^[19]^ This correlation may arise due to the influence of geographical factors. Fourth, differences in age, sex, dietary habits, and anti-*H pylori* IgG titers and the types and levels of anti-*H pylori* antibodies could also have a significant impact on the results.^[25]^ The above multiple factors lead to inconsistent results; therefore, the connection between *H pylori* and OP is still unclear and requires further research.

The rising burden of OP highlights the urgency to identify treatable factors for prevention and early intervention. MR was used to assess the causal association between contracting *H pylori* infection and OP. This approach offers several advantages over traditional observational studies. First, it effectively shields researchers from distorting the influence of confounding variables and reversing causation, thus leading to more reliable results. Second, by restricting our analysis to data from European populations, we minimized the potential biases stemming from environmental, racial, and dietary differences, thus further strengthening the study’s internal validity. In addition, we employed 5 methods to evaluate the causal connection that may exist between contracting *H pylori* and OP, and the results obtained using these 5 methods were similar. Simultaneously, the robustness of the results was verified using Cochran *Q* test, MR-Egger regression intercept test, and “leave-one-out” method.

This study has some limitations. First, the diagnosis of contracting *H pylori* used *H pylori* serology, which may have lacked accuracy. Second, this study only used samples from European populations and lacked samples from other regions, making it difficult to determine whether the results can be extended to other populations. In addition, the data used in this study lacked stratification, such as age, sex, and disease duration, which made it challenging to compare causal differences between subgroups. Therefore, larger-sample MR studies and randomized controlled trials are required to validate our results.

We employed a two-sample bidirectional MR method to explore the causal association between *H pylori* infection and OP. Our findings indicated no causal relationship between the 2, and we are convinced that our research offers meaningful conclusions regarding the prevention of OP.

## 5. Conclusions

This MR study was designed to explore the causal association between *H pylori* infection and the risk of OP. These results do not support the causal relationship between *H pylori* infection and OP.

## Acknowledgments

We would like to thank the participants and researchers involved in the FinnGen, EBI, and UK Biobank.

## Author contributions

**Conceptualization:** ZhiXiang Chen, Xiao Yang, Wen Zhang.

**Data curation:** ZhiXiang Chen, Xiao Yang, LuYao Wang, JianMin Fan.

**Formal analysis:** ZhiXiang Chen, Xiao Yang.

**Funding acquisition:** JianMin Fan, Wen Zhang.

**Project administration:** Wen Zhang.

**Writing – original draft:** ZhiXiang Chen, Xiao Yang, JianMin Fan.

**Writing – review & editing:** YaoWu Chen, MengLi Ji, MaoWen Wang, Wen Zhang.

## Supplementary Material




