# Supplementary material for: Bidirectional Mendelian randomization analysis reveals no causal association between Helicobacter pylori infection and osteoporosis risk
Source: Medicine (Baltimore). 2025 Oct 31;104(44):e45185. doi: 10.1097/MD.0000000000045185 (PMC12582754; doi:10.1097/MD.0000000000045185)
Supplement: Supplementary file 1 [file medi-104-e45185-s001.docx]

**Table S1**

Instrumental variants of anti Helicobacter pylori IgG seropositivity and F statistic

| SNPs | beta | se | pval | eaf | F |
| --- | --- | --- | --- | --- | --- |
| rs41263973 | 0.315775 | 0.0672338 | 2.74183E-06 | 0.033534 | 22.04929232 |
| rs2169557 | -0.105911 | 0.0230674 | 4.55229E-06 | 0.48846 | 21.07168801 |
| rs72708546 | -0.229692 | 0.0481386 | 1.90042E-06 | 0.059115 | 22.75722538 |
| rs35030589 | -0.175163 | 0.0342852 | 3.40432E-07 | 0.13205 | 26.09070764 |
| rs117912702 | 0.405025 | 0.0865994 | 3.01655E-06 | 0.019949 | 21.86494447 |
| rs73512476 | 0.212576 | 0.0440791 | 1.47479E-06 | 0.076735 | 23.24756698 |
| rs17502937 | -0.396711 | 0.0846646 | 2.89208E-06 | 0.020036 | 21.94619567 |
| rs74045808 | -0.17464 | 0.038012 | 4.48818E-06 | 0.10756 | 21.09893273 |
| rs78825412 | 0.31781 | 0.0685235 | 3.64158E-06 | 0.032426 | 21.50158746 |
| rs12591869 | -0.128595 | 0.0265143 | 1.28591E-06 | 0.26981 | 23.51268996 |
| rs55871438 | 0.29919 | 0.0647964 | 4.01976E-06 | 0.039419 | 21.31114948 |

Abbreviations: SNPs = Single Nucleotide Polymorphisms; EAF=effect allele frequency.

**Table S2**

Instrumental variants of H. pylori VacA antibodies and F statistic

| SNPs | beta | se | pval | eaf | F |
| --- | --- | --- | --- | --- | --- |
| rs72645538 | 0.752792 | 0.164377 | 4.65715E-06 | 0.011992 | 20.94665351 |
| rs113845906 | 0.413478 | 0.0845187 | 9.97401E-07 | 0.045066 | 23.90264467 |
| rs1530121 | -0.266111 | 0.0511414 | 1.95637E-07 | 0.86313 | 27.04127639 |
| rs77497849 | 0.264227 | 0.0538191 | 9.12935E-07 | 0.119223 | 24.07289294 |
| rs10246445 | -0.488144 | 0.106857 | 4.91972E-06 | 0.028197 | 20.84187156 |
| rs7019543 | 0.18107 | 0.0380994 | 2.00845E-06 | 0.320078 | 22.55815046 |
| rs117077218 | 0.640222 | 0.138755 | 3.94903E-06 | 0.016656 | 21.26232061 |
| rs9606224 | 0.507461 | 0.10223 | 6.90876E-07 | 0.029841 | 24.60908004 |
| rs133537 | -0.173609 | 0.0367088 | 2.25237E-06 | 0.615079 | 22.3383378 |

Abbreviations:*H. pylori* = *Helicobacter pylori;* VacA = Vacuolar cytotoxin A; SNPs = single-nucleotide polymorphism; EAF = effect allele frequency.

**Table S3**

Instrumental variants of H. pylori CagA antibodies and F statistic

| SNPs | beta | se | pval | eaf | F |
| --- | --- | --- | --- | --- | --- |
| rs75170215 | 0.743256 | 0.156416 | 2.01614E-06 | 0.02057 | 22.53366242 |
| rs75740599 | 0.235842 | 0.0512963 | 4.27307E-06 | 0.252886 | 21.09538736 |
| rs3998182 | 0.284762 | 0.0548049 | 2.03718E-07 | 0.27555 | 26.94279068 |
| rs4268452 | -0.384976 | 0.0840329 | 4.62189E-06 | 0.071203 | 20.94526676 |
| rs117827497 | -0.583731 | 0.125147 | 3.09585E-06 | 0.030462 | 21.7121043 |
| rs11858369 | 0.437721 | 0.0864978 | 4.18177E-07 | 0.068381 | 25.55653255 |
| rs118006294 | -0.475295 | 0.101815 | 3.03837E-06 | 0.050367 | 21.74804622 |

Abbreviations:*H. pylori* = *Helicobacter pylori*; CagA = cytotoxin-associated gene A protein ; SNPs = single nucleotide polymorphisms; EAF = effect allele frequency.

**Table S4**

Instrumental variants of OP and F statistic

| SNP | beta | se | pval | eaf | F |
| --- | --- | --- | --- | --- | --- |
| rs973825 | 0.1555 | 0.0332 | 2.83903E-06 | 0.8108 | 21.92367497 |
| rs12476059 | 0.2145 | 0.0449 | 1.80098E-06 | 0.09648 | 22.80818078 |
| rs6817223 | 0.1419 | 0.0279 | 3.57998E-07 | 0.6798 | 25.85146264 |
| rs7678610 | -0.1334 | 0.0269 | 7.35699E-07 | 0.3686 | 24.57739417 |
| rs75461305 | 0.1458 | 0.0319 | 4.70999E-06 | 0.2146 | 20.87672725 |
| rs3793233 | 0.2319 | 0.0463 | 5.425E-07 | 0.08818 | 25.07080337 |
| rs112312989 | 1.2524 | 0.2742 | 4.94094E-06 | 0.003032 | 20.84797104 |
| rs577348 | 0.1568 | 0.0287 | 4.469E-08 | 0.2909 | 29.8291516 |
| rs74555273 | 0.2015 | 0.0405 | 6.40206E-07 | 0.1174 | 24.73731918 |
| rs10795077 | 0.1328 | 0.0271 | 9.24507E-07 | 0.6419 | 23.9977291 |
| rs112213148 | 0.5064 | 0.1107 | 4.79999E-06 | 0.01366 | 20.91244108 |
| rs4085921 | 0.1211 | 0.0264 | 4.56604E-06 | 0.408 | 21.02775996 |
| rs1994535 | 0.1284 | 0.0265 | 0.00000128 | 0.6049 | 23.4621083 |
| rs11644631 | 0.1468 | 0.0289 | 3.71903E-07 | 0.2842 | 25.78511091 |
| rs144140959 | -0.3154 | 0.069 | 4.88799E-06 | 0.03791 | 20.88035002 |
| rs16940102 | -0.1229 | 0.026 | 2.31398E-06 | 0.5249 | 22.32901924 |
| rs2584298 | -0.1404 | 0.0307 | 4.86194E-06 | 0.2329 | 20.9011433 |
| rs117849041 | 0.1767 | 0.0376 | 2.68102E-06 | 0.1392 | 22.07038897 |
| rs4300899 | -0.2074 | 0.0452 | 4.38803E-06 | 0.09372 | 21.04036224 |

Abbreviations:OP = osteoporosis; SNPs = single nucleotide polymorphisms; EAF = effect allele frequency.

**Table S5**

MR estimates of assessing the bidirectional causal association between *H. pylori* and OP

| Exposure | Outcome | No.of SNPs | F-statistics | IVW | | MR-Egger regression | | MR-PRESSO |
| --- | --- | --- | --- | --- | --- | --- | --- | --- |
|  |  |  |  | Q statistic | Q_pval | Intercept | P_inter | P_global heterogeneity |
| H.pylori | OP | 11 | 246.45 | 7.35 | 0.69 | -0.008 | 0.82 | 0.72 |
| VacA | OP | 9 | 212.768 | 10.96 | 0.2 | 0.037 | 0.30 | 0.23 |
| CagA | OP | 7 | 160.53 | 6.15 | 0.41 | -0.089 | 0.15 | 0.42 |
| OP | H.pylori | 19 | 438.93 | 20.38 | 0.31 | -0.026 | 0.13 | 0.35 |

P_inter: P value for intercept test of multivariable MR-Egger

P_global heterogeneity: p value for global heterogeneity test of MR-PRESSO Abbreviations: SNPs = single nucleotide polymorphisms; IVW = inverse variance-weighted; *H. pylori* = *Helicobacter pylori*; VacA = vacuolar cytotoxin A; CagA =cytotoxin-associated gene A protein ; OP = osteoporosis.
