# Supplementary material for: Bidirectional Mendelian randomization analysis reveals no causal association between Helicobacter pylori infection and osteoporosis risk
Source: Medicine (Baltimore). 2025 Oct 31;104(44):e45185. doi: 10.1097/MD.0000000000045185 (PMC12582754; doi:10.1097/MD.0000000000045185)
Supplement: Supplementary file 2 [file medi-104-e45185-s002.docx]

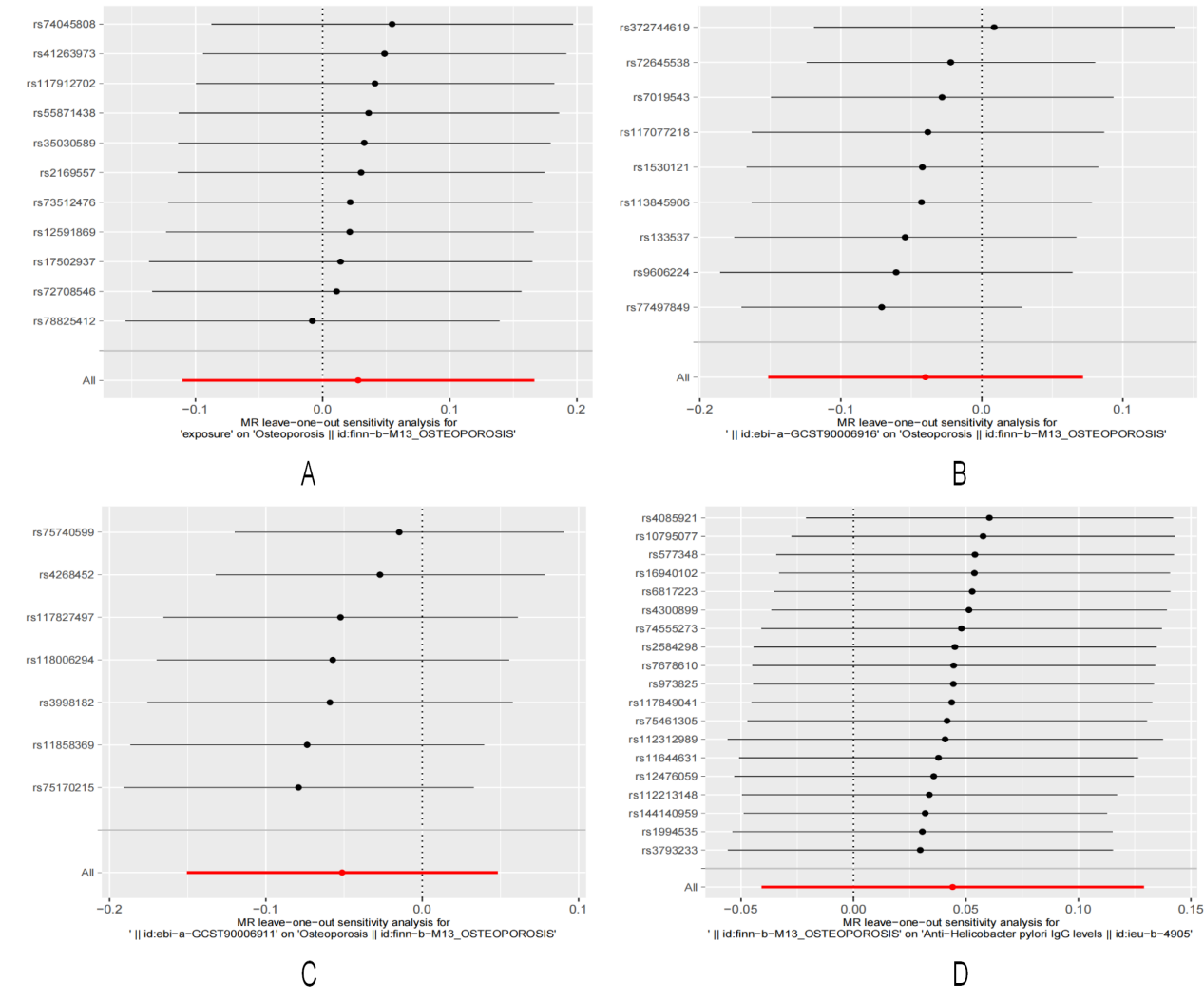
**Figure S1.** Leave-one-out plots.(A) Leave-one-out of SNPs associated with IgG and their risk of OP.(B) Leave-one-out of SNPs associated with VacA and their risk of OP.(C) Leave-one-out of SNPs associated with CagA and their risk of OP.(D) Leaveone-out of SNPs associated with OP and their risk of Hp.


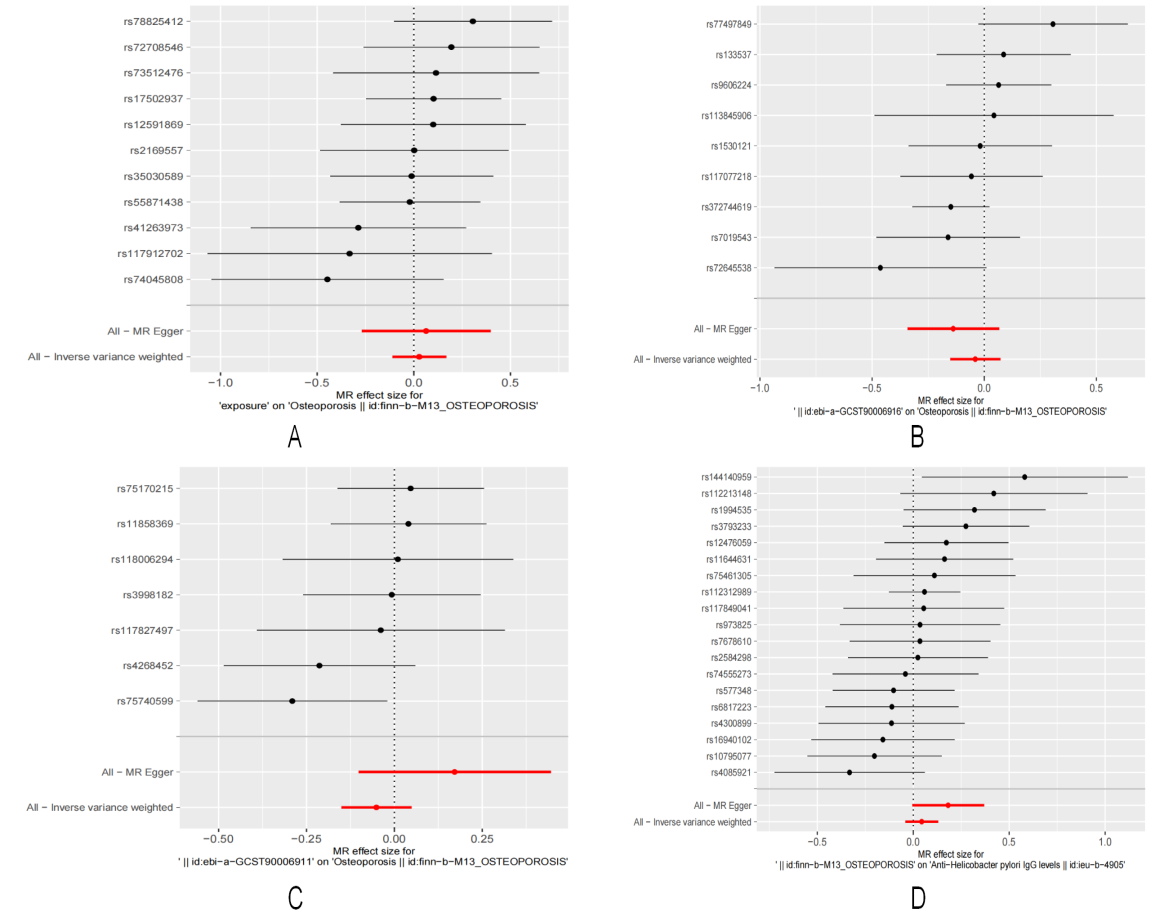
**Figure S2.** Forest plots.(A) Forest of SNPs associated with IgG and their risk of OP.(B) Forest of SNPs associated with VacA and their risk of OP.(C) Forest of SNPs associated with CagA and their risk of OP.(D) Forest of SNPs associated with OP and their risk of Hp.


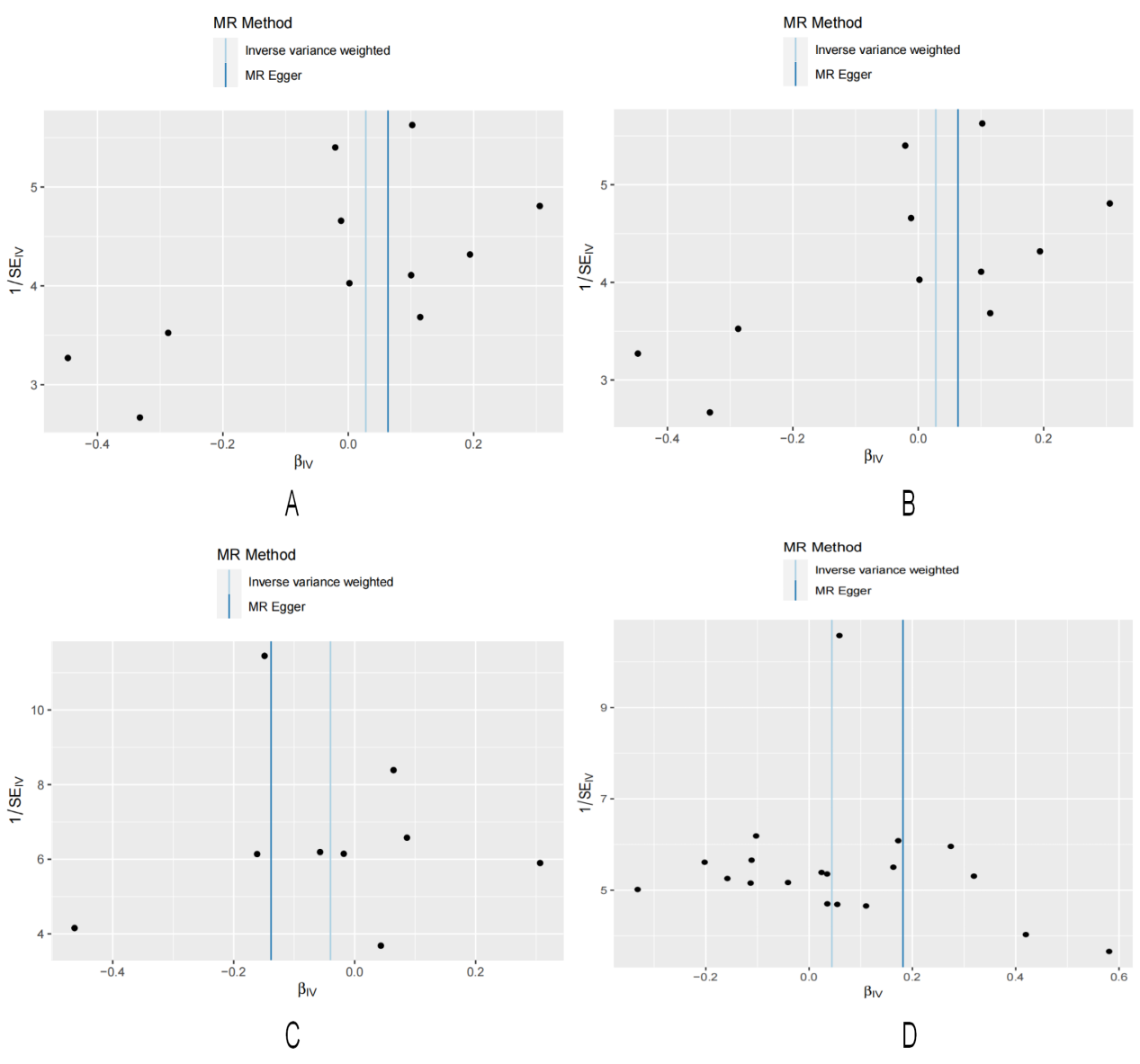
**Figure S3.**Funnel plots.(A) Funnel of SNPs associated with IgG and their risk of OP.(B) Funnel of SNPs associated with VacA and their risk of OP.(C) Funnel of SNPs associated with CagA and their risk of OP.(D) Funnel of SNPs associated with OP and their risk of Hp.
